# Clinical trial management: a profession in crisis?

**DOI:** 10.1186/s13063-022-06315-8

**Published:** 2022-04-27

**Authors:** E. J. Mitchell, K. Goodman, N. Wakefield, C. Cochran, S. Cockayne, S. Connolly, R. Desai, S. Hartley, S. A. Lawton, K. Oatey, S. Rhodes, J. S. Savage, J. Taylor, N. F. J. Youssouf, on behalf of the UK Trial Managers’ Network, Eleanor Mitchell, Kirsteen Goodman, Natalie Wakefield, Claire Cochran, Sarah Cockayne, Simon Connolly, Riti Desai, Lucy Fletcher, Suzanne Hartley, Sarah Lawton, Ryonfa Lee, Katherine Oatey, Shelley Rhodes, Joshua Savage, Jodi Taylor, Nabila Youssouf

**Affiliations:** 1 Nottingham Clinical Trials Unit, School of Medicine, University of Nottingham, University Park, Nottingham, NG7 2RD UK; 2 NMAHP Research Unit, Glasgow Caledonian University, Glasgow, G4 0NA UK; 3 Centre for Healthcare and Randomised Controlled Trials (CHaRT), Health Services Research Unit, University of Aberdeen, Aberdeen, AB23 2ZD UK; 4 York Trials Unit, Department of Health Sciences, University of York, York, YO10 5DD UK; 5 Royal Marsden Clinical Trials Unit, The Royal Marsden NHS Foundation Trust, London, SW3 6JJ UK; 6 King’s Ophthalmology Research Unit, King’s College Hospital, London, SE5 9RS UK; 7 Clinical Trials Research Unit, Leeds Institute of Clinical Trials Research, University of Leeds, Leeds, LS2 9JT UK; 8 Keele Clinical Trials Unit, School of Medicine, Keele University, Keele, ST5 5BG UK; 9 Edinburgh Clinical Trials Unit, University of Edinburgh, Edinburgh, EH16 4UX UK; 10 Exeter Clinical Trials Unit, University of Exeter, St Luke’s Campus, Exeter, EX1 2LU UK; 11 Cancer Research UK Clinical Trials Unit, Institute of Cancer and Genomic Sciences, University of Birmingham, Birmingham, B15 2TT UK; 12 Bristol Randomised Trials Collaboration, Bristol Trials Centre, University of Bristol, 39 Whatley Road, Bristol, BS8 2PS UK; 13 Clinical Research Department, London School of Hygiene and Tropical Medicine, Keppel Street, London, WC1E 7HT UK

## Abstract

Clinical trial managers play a vital role in the design and conduct of clinical trials in the UK. There is a current recruitment and retention crisis for this specialist role due to a complex set of factors, most likely to have come to a head due to the COVID-19 pandemic. Academic clinical trial units and departments are struggling to recruit trial managers to vacant positions, and multiple influences are affecting the retention of this highly skilled workforce. Without tackling this issue, we face major challenges in the delivery on the Department of Health and Social Care’s Future of UK Clinical Research Delivery implementation plan. This article, led by a leading network of and for UK Trial Managers, presents some of the issues and ways in which national stakeholders may be able to address this.

## Background

Like many employment sectors, the clinical trial workforce has been substantially affected in recent years, most likely due to the COVID-19 pandemic. Trial management professionals play a pivotal role in the design and conduct of clinical trials and are vital members of the research team. For brevity, we refer to trial management professionals as trial managers throughout this article. However, we include a breadth of roles in this discipline, recognising the huge variety of job titles in the UK. The specialist role of a trial manager is essential to the successful delivery of clinical trials [1–3], yet the clinical trial management profession is currently experiencing a recruitment and retention crisis. Many clinical trials units and departments that conduct academic, non-commercial trials, across the UK, are struggling to recruit trial managers to vacant positions and, in addition, experienced trial managers are leaving the profession. At the time of writing (November 2021), there are currently 107 trial management positions being advertised on www.jobs.ac.uk, the recruitment website commonly used for advertising vacancies in academia.

The Department for Health and Social Care’s (DHSC) recently published “Future of UK Clinical Research Delivery” implementation plan [4] focuses on having a sustained and supported research workforce in order for the UK to conduct world-leading clinical research: it is therefore essential that this issue is tackled urgently. The UK Trial Managers’ Network (UKTMN) is a national network, currently with ~1400 trial management professionals as members, and has been involved in numerous discussions with trial management teams and senior staff based in UK clinical trial units (CTUs) about the problem of recruiting and retaining trial managers, which has prompted this article. In this article, we describe the problem and offer some solutions.

### Problems

#### Lack of identity and support

Clinical trial management is a highly rewarding, specialist, skilled role, essential for the design and conduct of high-quality clinical trials. Unfortunately, however, the role has not been recognised and valued in the past, with some viewing it as a supporting, administrative function within clinical trials. In many organisations, trial managers are employed either on a ‘research’ or ‘professional support services’ career pathway, neither of which fully suit the role, which is often a hybrid of the two. The lack of recognition and appropriate career pathway in many organisations for this vital, specialist role, has led to trial managers feeling undervalued [2].

#### Changes to ways of working

The acceleration in offering more flexible working arrangements by universities and NHS Trusts, together with access to technology allowing virtual working, arisen as a result of the COVID-19 pandemic, has led to trial managers having a wider choice of where they are based, to utilise their expertise. Indeed, many trial managers are moving between universities, rather than being bound by geographical constraints of a more local university. Where geography may have previously limited employment options, trial managers can now consider applying for similar positions further afield, within or outside of academia, and work flexibly, without the need for 100% office-based working. The pandemic may have also changed people’s priorities with respect to their working arrangements and work-life balance: a flexible approach to working is often highly valued by many individuals, particularly those who may also have caring responsibilities or a long commute in their previous roles.

#### UK research pipeline

The pause on non-COVID-19-related clinical trials during 2020 and continuing into 2021 has had a knock-on effect on the research pipeline in many organisations, with many clinical trial units and departments now having many clinical trials in the set-up stage, which is hugely time and resource intensive. Furthermore, due to the pause of many clinical trials during the pandemic and subsequent slow restart of some trials, overall, many departments’ trial portfolios are larger and, in some cases, there are more trials needing management than the number of trial managers available. In December 2019 (pre-pandemic), the National Institute for Health Research had 1336 active projects across their portfolio. In comparison, there were 2026 active projects across the portfolio on 31 December 2021: an increase of 52% in just 2 years [5].

With many clinical trial manager positions currently vacant, the knock-on effect to the remaining trial management workforce has been huge, and the fast pace of change has created instability and uncertainties within the profession. With a reduced workforce, remaining clinical trial managers are tasked with managing additional clinical trials, thus contributing to an increase in workload, and trial management staff expressing concerns about burnout (personal communications). An increase in workload, whilst ensuring the work-life balance of staff, may be possible in the short term, but is not a sustainable option for the future. If the situation is not addressed, the profession will face a vicious cycle, with the knock-on effect of trial managers leaving positions then creating further instability and more people choosing to look for alternative positions. Furthermore, losing the expertise of clinical trial managers places an additional burden and resource implications in training new staff.

It is a long-standing perennial issue that academic trials are unable to compete in offering similar financial remuneration, rewards and job security to those offered by the commercial sector. Despite this, we must address ways in which academic trials are able to offer a supportive, flexible, environment with recognition and reward and good career prospects, in order to retain the highly skilled and experienced trial managers of today and attract and recruit trial managers for tomorrow. Without the workforce in place to deliver the ambitions outlined in the DHSC vision, the plan will fail.

### Potential strategies to overcome the problems

#### Professional development and recognition of the role

Theme five of the DHSC's future vision is welcomed: “a sustainable and supported research workforce – which offers opportunities and exciting careers for all healthcare and research staff of all professional backgrounds”*.* Supporting the professional development of clinical trial managers is essential if we are to retain the workforce. Employers of clinical trial managers must invest time and effort into supporting development and promotion opportunities, including training, and recognise the role as highly valuable and important to the UK research landscape. Having the time and funding to access training opportunities is considered the most important aspect of professional development for clinical trial managers [6]. The creation of opportunities, by funding bodies, for trial managers to receive training, similar to opportunities that exist for other clinical trial disciplines, would be well received. High value is also placed upon having the opportunity to be involved in activities outside of the day-to-day running of a clinical trial, in particular being involved in aspects of trial design, and undertaking qualifications relevant to the role. UKTMN has recently published its professional development strategy with a range of initiatives planned to support clinical trial managers and increase recognition in the role [7]. As outlined in this strategy, a key area of focus is exploring alternative job families/career pathways, due to the hybrid, specialist nature of the role, which neither fits research nor professional service pathways. We encourage CTUs and departments to open up discussions with employers about offering alternative pathways, for the many multidisciplinary roles required to design and deliver clinical trials and other research studies, including trial managers, within their institutions. Employers and line managers of clinical trial managers are also encouraged to utilise the freely available UKTMN Competency Framework which can be used to support appraisals and professional development discussions [8]. Finally, the substantial contribution a trial manager makes to a clinical trial should be recognised via authorship on clinical trial publications and whilst some progress has been made in this area, recognition of trial managers is inconsistent.

#### Flexible working and fixed-term contracts

To retain a highly valued, specialist workforce, it is essential for employers of trial managers of academic trials to consider flexible working options, including home-based working, similar to what has been offered by commercial contract research organisations and pharmaceutical companies for many years. Given that a significant proportion of trial managers are female (~90%) [9], who may also be the primary carer for children, this is particularly important to pay attention to. Employers may wish to consider offering fully remote roles, where appropriate, though this also needs balancing with the need for training, team-working and social aspects of working collaboratively in the same location. This is particularly important to consider for new staff starting, who may miss the informal support often available from office-based working, and formal training required within the role. Though flexible and remote working options should be offered, some activities, particularly training of new trial managers, may be required to be in-person, though virtual or hybrid models of training should also be considered. The onset of affordable and secure electronic platforms for remote yet compliant trial management can enable a flexible approach to working, both in terms of working hours, working times and location, in line with the operational needs of the clinical trial. Moving forwards, financial support for these electronic systems must be fully costed into funding applications. This relative cost-effective strategy may help address competing demands and go some way to achieving an improved work-life balance for trial managers. We do recognise, however, that not all trial managers may wish to work remotely and flexibly and that this way of working can also present challenges, particularly to trial managers working in large teams. If possible, flexibility *and* choice should be offered, leading to an empowered and satisfied workforce.

Where possible, offering permanent or longer-term contracts is important and employers must consider this. Previous survey work of trial managers found that ‘funding’ and ‘fixed-term contracts’ were substantial barriers to career development [6]. Indeed, of the 107 roles advertised on jobs.ac.uk. at the time of writing, 72 (67%) were advertised as having a fixed-term contract, of which 28 (39%) offered a contract of ≤12 months and 22 (31%) were for ≤24 months.

#### Attracting trial managers of the future

Managing non-commercial clinical trials is a challenging but rewarding role. Whilst job descriptions differ between organisations, many of the tasks and activities are similar and wide-ranging, though salary ranges differ greatly depending upon the level of seniority of a trial management position. The role can offer opportunities to achieve a great sense of ownership of a clinical trial, team and collaborative working and utilisation of complex project management and problem-solving skills, which may benefit career progression. In many non-commercial clinical trials, the trial management role encompasses a wide variety of activities, which is valued by the workforce since the varied role can provide job satisfaction. The wide range of specialist skills developed in the role is highly valuable to UK research. Clinical trial management tasks may also include involvement in, or leading, additional projects, such as methodology studies to address gaps in trial methodology evidence. The UK Trial Managers’ Network (UKTMN) provides a wide range of example job descriptions on its website [10]. To attract new trial managers to the profession, clarity on the importance of the role, the varied job description and a clearly defined career pathway will be important to publicise, outside of the existing academic clinical trial infrastructure. Employers should carefully consider how these positions are advertised in order to attract new people to the profession, for example, advertising beyond academic websites (including social media platforms such as LinkedIn) and promoting trial management careers through undergraduate and postgraduate courses. Promoting careers in clinical trials at job fairs and career events and via open days is also recommended — many people may have transferable skills which could be well suited to the trial management profession. Offering more ‘entry-level’ positions, so that staff can gain experience ‘on the job’, could also be helpful for building a sustainable trial management workforce.

## Conclusion

In summary, clinical trial managers are key to the UK clinical trial delivery capacity and a range of solutions are required to tackle the increasing problem of recruitment and retention of this specialist workforce. Due to the complexity of the issue, a national collaborative effort, from a range of stakeholders, is needed. If we are unable to recruit and retain trial managers, delays will occur in studies which can impact upon further funding and ultimately delay answering important healthcare questions. Investing in clinical trial managers, and tackling this issue, is urgently needed if we are to achieve the vision outlined by the Department for Health and Social care earlier this year.

## Data Availability

Data sharing is not applicable to this article as no datasets were generated or analysed during the current study.
